# Supplementary material for: Prognostic Impacts of LL-37 in Relation to Lipid Profiles of Patients with Myocardial Infarction: A Prospective Cohort Study
Source: Biomolecules. 2022 Oct 14;12(10):1482. doi: 10.3390/biom12101482 (PMC9599865; doi:10.3390/biom12101482)

Supplementary

Material

Supplementary Table S1. Univariable analysis for major adverse cardiovascular events.

Variables	HR (95 % CI)	P-value
Age, years	1.04 (1.03–1.05)	<0.001
Male sex	0.72 (0.54–0.96)	0.024
BMI, kg/m^2	0.95 (0.91–0.98)	0.004
Hypertension	1.57 (1.18–2.08)	0.002
Diabetes	1.19 (0.92–1.54)	0.176
Smoking	0.96 (0.74–1.26)	0.788
Previous myocardial infarction	1.81 (1.37–2.40)	<0.001
Heart rate, bpm	1.01 (1.00–1.02)	0.004
Systolic blood pressure, mmHg	1.00 (0.99–1.01)	0.277
Diastolic blood pressure, mmHg	1.00 (0.99–1.01)	0.574
EF, %	0.95 (0.94–0.96)	<0.001
Leucocytes, ×10^9/L	1.03 (0.99–1.07)	0.073
Hemoglobin, g/L	0.99 (0.98–0.99)	<0.001
Platelets, ×10^9/L	1.00 (0.99–1.00)	0.109
Creatinine, µmol/L	1.01 (1.00–1.01)	<0.001
Glucose, mmol/L	1.04 (1.02–1.08)	0.001
HbA1c, %	1.10 (1.02–1.18)	0.010
HsCRP, mg/L	1.04 (1.02–1.06)	<0.001
cTnI, ng/mL	1.01 (1.00–1.02)	0.001
NT-proBNP, per 100-pg/mL	1.01 (1.01–1.01)	<0.001
LDL-C, mmol/L	0.76 (0.66–0.88)	<0.001
HDL-C, mmol/L	1.13 (0.81–1.57)	0.469
Lp(a), mg/L	1.00 (0.99–1.00)	0.921
Triglyceride, mmol/L	0.93 (0.83–1.05)	0.239
LL-37 (high vs low)	0.71 (0.55–0.91)	0.007
PCSK9, ng/mL	1.00 (0.99–1.00)	0.212
Culprit lesion		
LM	2.97 (1.21–7.31)	0.018
LAD	0.96 (0.72–1.26)	0.755
LCX	0.97 (0.66–1.43)	0.882
RCA	1 (reference)	–
Bypass graft	4.69 (2.18–10.11)	<0.001
Multivessel disease		
1-vessel disease	1 (reference)	–
2-vessel disease	1.38 (0.95–2.01)	0.091
3-vessel disease	1.81 (1.30–2.54)	0.001
Successful revascularizations	0.35 (0.26–0.46)	<0.001
Aspirin	0.68 (0.40–1.17)	0.169
P2Y12 inhibitor	0.37 (0.18–0.75)	0.006
Statin	0.60 (0.37–0.98)	0.041

BMI = body mass index, CI = confidence interval, cTnI = cardiac troponin I, EF = ejection fraction, HbA1c = hemoglobin A1c, HDL-C = high-density lipoprotein cholesterol, hsCRP = high sensitivity C-reactive protein, HR = hazard ratio, LAD = left anterior descending artery, LCX = left circumflex, LDL-C = low-density lipoprotein cholesterol, LM = left main, lp(a) = lipoprotein (a), NT-proBNP = N-terminal prohormone of brain natriuretic peptide, PCSK9 = proprotein convertase subtilisin/kexin type 9, RCA = right coronary artery, STEMI = ST-segment elevation myocardial infarction.

Supplementary Table S2. Univariable analysis for the composite of cardiac death, recurrent myocardial infarction and ischemic stroke.

Variables	HR (95 % CI)	P-value
Age, years	1.03 (1.01–1.04)	<0.001
Male sex	0.85 (0.61–1.19)	0.342
BMI, kg/m^2	0.95 (0.91–0.98)	0.006
Hypertension	1.50 (1.10–2.03)	0.010
Diabetes	1.19 (0.90–1.58)	0.223
Smoking	1.00 (0.74–1.34)	0.979
Previous myocardial infarction	2.13 (1.58–2.87)	<0.001
Heart rate, bpm	1.01 (1.00–1.02)	0.060
Systolic blood pressure, mmHg	1.00 (1.00–1.01)	0.280
Diastolic blood pressure, mmHg	1.00 (0.99–1.01)	0.887
EF, %	0.95 (0.94–0.97)	<0.001
Leucocytes, ×10^9/L	1.00 (0.96–1.05)	0.921
Hemoglobin, g/L	0.99 (0.98–1.00)	0.001
Platelets, ×10^9/L	1.00 (1.00–1.00)	0.258
Creatinine, μmol/L	1.00 (1.00–1.01)	<0.001
Glucose, mmol/L	1.04 (1.01–1.07)	0.016
HbA1c, %	1.09 (1.01–1.18)	0.029
HsCRP, mg/L	1.02 (1.00–1.05)	0.075
cTnI, ng/mL	1.01 (1.00–1.01)	0.001
NT-proBNP, per 100-pg/mL	1.01 (1.01–1.01)	<0.001
LDL-C, mmol/L	0.76 (0.65–0.89)	0.001
HDL-C, mmol/L	1.12 (0.78–1.62)	0.526
Lp(a), mg/L	1.00 (1.00–1.00)	0.249
Triglyceride, mmol/L	0.96 (0.85–1.09)	0.522
LL-37 (high vs low)	0.70 (0.53–0.92)	0.010
PCSK9, ng/mL	1.00 (1.00–1.00)	0.486
Culprit lesion		
LM	2.06 (0.65–6.54)	0.218
LAD	0.94 (0.70–1.27)	0.679
LCX	0.94 (0.61–1.44)	0.771
RCA	1 (reference)	–
Bypass graft	1.59 (0.39–6.45)	0.520
Multivessel disease		
1-vessel disease	1 (reference)	–
2-vessel disease	1.28 (0.85–1.94)	0.235
3-vessel disease	1.78 (1.23–2.57)	0.002
Successful revascularizations	0.33 (0.24–0.45)	<0.001
Aspirin	0.89 (0.45–1.73)	0.720
P2Y12 inhibitor	0.35 (0.16–0.74)	0.006
Statin	0.65 (0.37–1.13)	0.128

BMI = body mass index, CI = confidence interval, cTnI = cardiac troponin I, EF = ejection fraction, HbA1c = hemoglobin A1c, HDL-C = high-density lipoprotein cholesterol, hsCRP = high sensitivity C-reactive protein, HR = hazard ratio, LAD = left anterior descending artery, LCX = left circumflex, LDL-C = low-density lipoprotein cholesterol, LM = left main, lp(a) = lipoprotein (a), NT-proBNP = N-terminal prohormone of brain natriuretic peptide, PCSK9 = proprotein convertase subtilisin/kexin type 9, RCA = right coronary artery, STEMI = ST-segment elevation myocardial infarction.

Supplementary Table S3. Univariable analysis for all-cause death.

Variables	HR (95 % CI)	P-value
Age, years	1.07 (1.06–1.09)	<0.001
Male sex	0.49 (0.33–0.73)	<0.001
BMI, kg/m^2	0.90 (0.85–0.95)	<0.001
Hypertension	1.83 (1.18–2.84)	0.007
Diabetes	1.44 (0.99–2.09)	0.057
Smoking	0.81 (0.55–1.20)	0.288
Previous myocardial infarction	1.68 (1.10–2.56)	0.016
Heart rate, bpm	1.03 (1.02–1.04)	<0.001
Systolic blood pressure, mmHg	0.99 (0.98–1.00)	0.075
Diastolic blood pressure, mmHg	0.98 (0.96–0.99)	0.002
EF, %	0.92 (0.90–0.93)	<0.001
Leucocytes, ×10^9/L	1.13 (1.08–1.18)	<0.001
Hemoglobin, g/L	0.98 (0.97–0.98)	<0.001
Platelets, ×10^9/L	1.00 (1.00–1.00)	0.272
Creatinine, μmol/L	1.01 (1.00–1.01)	<0.001
Glucose, mmol/L	1.10 (1.06–1.14)	<0.001
HbA1c, %	1.17 (1.06–1.29)	0.003
HsCRP, mg/L	1.07 (1.05–1.10)	<0.001
cTnI, ng/mL	1.01 (1.01–1.03)	<0.001
NT-proBNP, per 100-pg/mL	1.01 (1.01–1.02)	<0.001
LDL-C, mmol/L	0.71 (0.58–0.89)	0.002
HDL-C, mmol/L	1.17 (0.73–1.89)	0.519
Lp(a), mg/L	1.00 (1.00–1.00)	0.567
Triglyceride, mmol/L	0.76 (0.61–0.95)	0.017
LL-37 (high vs low)	0.66 (0.46–0.96)	0.032
PCSK9, ng/mL	1.00 (1.00–1.00)	0.327
Culprit lesion		
LM	6.14 (2.19–17.25)	0.001
LAD	1.14 (0.75–1.75)	0.353
LCX	1.08 (0.59–1.96)	0.805
RCA	1 (reference)	–
Bypass graft	10.58 (4.46–25.07)	<0.001
Multivessel disease		
1-vessel disease	1 (reference)	–
2-vessel disease	1.96 (1.02–3.78)	0.043
3-vessel disease	3.20 (1.77–5.79)	<0.001
Successful revascularizations	0.32 (0.21–0.48)	<0.001
Aspirin	0.37 (0.20–0.70)	0.002
P2Y12 inhibitor	0.29 (0.12–0.70)	0.007
Statin	0.36 (0.20–0.65)	0.001

BMI = body mass index, CI = confidence interval, cTnI = cardiac troponin I, EF = ejection fraction, HbA1c = hemoglobin A1c, HDL-C = high-density lipoprotein cholesterol, hsCRP = high sensitivity C-reactive protein, HR = hazard ratio, LAD = left anterior descending artery, LCX = left circumflex, LDL-C = low-density lipoprotein cholesterol, LM = left main, lp(a) = lipoprotein (a), NT-proBNP = N-terminal prohormone of brain natriuretic peptide, PCSK9 = proprotein convertase subtilisin/kexin type 9, RCA = right coronary artery, STEMI = ST-segment elevation myocardial infarction.

Supplementary Table S4. Univariable analysis for cardiac death.

Variables	HR (95 % CI)	P-value
Age, years	1.06 (1.04–1.08)	<0.001
Male sex	0.54 (0.31–0.92)	0.024
BMI, kg/m^2	0.85 (0.79–0.92)	<0.001
Hypertension	1.75 (0.98–3.12)	0.059
Diabetes	1.54 (0.94–2.53)	0.088
Smoking	0.71 (0.42–1.18)	0.182
Previous myocardial infarction	2.80 (1.68–4.68)	<0.001
Heart rate, bpm	1.04 (1.02–1.05)	<0.001
Systolic blood pressure, mmHg	0.98 (0.97–0.99)	0.012
Diastolic blood pressure, mmHg	0.97 (0.95–0.99)	0.006
EF, %	0.90 (0.87–0.92)	<0.001
Leucocytes, ×10^9/L	1.12 (1.06–1.20)	<0.001
Hemoglobin, g/L	0.98 (0.97–0.99)	<0.001
Platelets, ×10^9/L	1.00 (1.00–1.00)	0.845
Creatinine, μmol/L	1.01 (1.00–1.01)	<0.001
Glucose, mmol/L	1.11 (1.06–1.16)	<0.001
HbA1c, %	1.18 (1.03–1.34)	0.015
HsCRP, mg/L	1.06 (1.03–1.10)	0.001
cTnI, ng/mL	1.02 (1.01–1.03)	<0.001
NT-proBNP, per 100-pg/mL	1.01 (1.01–1.02)	<0.001
LDL-C, mmol/L	0.68 (0.51–0.91)	0.010
HDL-C, mmol/L	1.28 (0.73–2.26)	0.385
Lp(a), mg/L	1.00 (1.00–1.01)	0.019
Triglyceride, mmol/L	0.79 (0.60–1.05)	0.106
LL-37 (high vs low)	0.63 (0.38–1.04)	0.070
PCSK9, ng/mL	1.00 (0.99–1.00)	0.735
Culprit lesion		
LM	5.46 (1.27–23.37)	0.022
LAD	1.29 (0.74–2.26)	0.375
LCX	1.18 (0.54–2.59)	0.680
RCA	1 (reference)	–
Bypass graft	3.28 (0.44–24.42)	0.247
Multivessel disease		
1-vessel disease	1 (reference)	–
2-vessel disease	2.46 (0.88–6.82)	0.084
3-vessel disease	5.09 (2.02–12.84)	0.001
Successful revascularizations	0.26 (0.15–0.44)	<0.001
Aspirin	0.46 (0.19–1.16)	0.100
P2Y12 inhibitor	0.27 (0.08–0.85)	0.025
Statin	0.41 (0.18–0.95)	0.037

BMI = body mass index, CI = confidence interval, cTnI = cardiac troponin I, EF = ejection fraction, HbA1c = hemoglobin A1c, HDL-C = high-density lipoprotein cholesterol, hsCRP = high sensitivity C-reactive protein, HR = hazard ratio, LAD = left anterior descending artery, LCX = left circumflex, LDL-C = low-density lipoprotein cholesterol, LM = left main, lp(a) = lipoprotein (a), NT-proBNP = N-terminal prohormone of brain natriuretic peptide, PCSK9 = proprotein convertase subtilisin/kexin type 9, RCA = right coronary artery, STEMI = ST-segment elevation myocardial infarction.

Supplementary Table S5. Univariable analysis for recurrent myocardial infarction.

Variables	HR (95 % CI)	P-value
Age, years	1.02 (0.99–1.03)	0.071
Male sex	1.12 (0.64–1.96)	0.683
BMI, kg/m²	0.99 (0.93–1.05)	0.717
Hypertension	1.17 (0.74–1.84)	0.485
Diabetes	0.71 (0.45–1.14)	0.159
Smoking	1.15 (0.72–1.85)	0.559
Previous myocardial infarction	2.17 (1.38–3.41)	0.001
Heart rate, bpm	0.99 (0.97–1.00)	0.165
Systolic blood pressure, mmHg	1.01 (0.99–1.02)	0.089
Diastolic blood pressure, mmHg	1.00 (0.99–1.02)	0.613
EF, %	0.99 (0.96–1.02)	0.457
Leucocytes, ×10⁹/L	0.94 (0.88–1.02)	0.122
Hemoglobin, g/L	0.99 (0.98–1.00)	0.206
Platelets, ×10⁹/L	1.00 (1.00–1.00)	0.137
Creatinine, μmol/L	1.00 (1.00–1.01)	0.046
Glucose, mmol/L	1.00 (0.95–1.06)	0.928
HbA1c, %	0.98 (0.85–1.12)	0.735
HsCRP, mg/L	0.99 (0.95–1.04)	0.748
cTnI, ng/mL	1.00 (0.99–1.01)	0.913
NT-proBNP, per 100-pg/mL	1.01 (1.00–1.01)	0.018
LDL-C, mmol/L	0.81 (0.64–1.02)	0.078
HDL-C, mmol/L	0.88 (0.45–1.73)	0.711
Lp(a), mg/L	1.00 (1.00–1.00)	0.751
Triglyceride, mmol/L	1.06 (0.90–1.24)	0.494
LL-37 (high vs low)	0.81 (0.53–1.24)	0.338
PCSK9, ng/mL	1.00 (0.98–1.01)	0.673
Culprit lesion		
LM	1.63 (0.22–11.89)	0.631
LAD	0.90 (0.57–1.42)	0.649
LCX	0.98 (0.52–1.85)	0.946
RCA	1 (reference)	–
Bypass graft	1.87 (0.26–13.67)	0.536
Multivessel disease		
1-vessel disease	1 (reference)	–
2-vessel disease	1.12 (0.61–2.04)	0.715
3-vessel disease	1.40 (0.82–2.39)	0.220
Successful revascularizations	0.42 (0.25–0.70)	0.001
Aspirin	1.78 (0.44–7.25)	0.419
P2Y12 inhibitor	0.56 (0.14–2.26)	0.413
Statin	0.90 (0.33–2.46)	0.839

BMI = body mass index, CI = confidence interval, cTnI = cardiac troponin I, EF = ejection fraction, HbA1c = hemoglobin A1c, HDL-C = high-density lipoprotein cholesterol, hsCRP = high sensitivity C-reactive protein, HR = hazard ratio, LAD = left anterior descending artery, LCX = left circumflex, LDL-C = low-density lipoprotein cholesterol, LM = left main, lp(a) = lipoprotein (a), NT-proBNP = N-terminal prohormone of brain natriuretic peptide, PCSK9 = proprotein convertase subtilisin/kexin type 9, RCA = right coronary artery, STEMI = ST-segment elevation myocardial infarction.

Supplementary Table S6. Univariable analysis for ischemic stroke.

Variables	HR (95 % CI)	P-value
Age, years	1.01 (0.99–1.03)	0.194
Male sex	0.94 (0.52–1.73)	0.852
BMI, kg/m^2	0.93 (0.87–1.00)	0.058
Hypertension	2.09 (1.16–3.77)	0.014
Diabetes	2.02 (1.25–3.26)	0.004
Smoking	1.11 (0.64–1.89)	0.725
Previous myocardial infarction	1.65 (0.95–2.86)	0.075
Heart rate, bpm	1.00 (0.99–1.02)	0.884
Systolic blood pressure, mmHg	1.01 (1.00–1.02)	0.033
Diastolic blood pressure, mmHg	1.01 (0.99–1.03)	0.057
EF, %	0.97 (0.94–0.99)	0.043
Leucocytes, ×10^9/L	0.94 (0.86–1.02)	0.143
Hemoglobin, g/L	1.00 (0.98–1.01)	0.457
Platelets, ×10^9/L	1.00 (1.00–1.00)	0.563
Creatinine, μmol/L	1.00 (1.00–1.00)	0.343
Glucose, mmol/L	1.05 (0.99–1.11)	0.079
HbA1c, %	1.18 (1.04–1.34)	0.009
HsCRP, mg/L	1.02 (0.97–1.06)	0.519
cTnI, ng/mL	1.01 (1.00–1.02)	0.176
NT-proBNP, per 100-pg/mL	1.01 (1.00–1.01)	0.013
LDL-C, mmol/L	0.76 (0.57–1.00)	0.049
HDL-C, mmol/L	1.31 (0.76–2.25)	0.332
Lp(a), mg/L	1.00 (1.00–1.00)	0.298
Triglyceride, mmol/L	0.90 (0.71–1.14)	0.376
LL-37 (high vs low)	0.63 (0.38–1.02)	0.060
PCSK9, ng/mL	1.00 (1.00–1.00)	0.208
Culprit lesion		
LM	0.83 (0.49–1.38)	0.464
LAD	0.82 (0.39–1.72)	0.594
LCX	–	–
RCA	1 (reference)	–
Bypass graft	–	–
Multivessel disease		
1-vessel disease	1 (reference)	–
2-vessel disease	1.30 (0.67–2.50)	0.434
3-vessel disease	1.18 (0.63–2.19)	0.605
Successful revascularizations	0.44 (0.24–0.79)	0.006
Aspirin	0.86 (0.27–2.73)	0.794
P2Y12 inhibitor	0.39 (0.10–1.60)	0.193
Statin	0.92 (0.29–2.91)	0.881

BMI = body mass index, CI = confidence interval, cTnI = cardiac troponin I, EF = ejection fraction, HbA1c = hemoglobin A1c, HDL-C = high-density lipoprotein cholesterol, hsCRP = high sensitivity C-reactive protein, HR = hazard ratio, LAD = left anterior descending artery, LCX = left circumflex, LDL-C = low-density lipoprotein cholesterol, LM = left main, lp(a) = lipoprotein (a), NT-proBNP = N-terminal prohormone of brain natriuretic peptide, PCSK9 = proprotein convertase subtilisin/kexin type 9, RCA = right coronary artery, STEMI = ST-segment elevation myocardial infarction.

Supplementary Table S7. Associations between LL-37 and clinical outcomes stratified by lipid profiles.

	MACE HR (95 % CI)	P-value	P int	Cardiac death, re-MI and ischemic stroke HR (95 % CI)	P-value	P int	All-cause death HR (95 % CI)	P-value	P int	Cardiac death HR (95 % CI)	P-value	P int	Re-MI HR (95 % CI)	P-value	P int	Ischemic stroke HR (95 % CI)	P-value	P int
Lp(a)																		
Lp(a) ≥ 300 mg/L	0.44 (0.25–0.79)	0.006	0.026	0.43 (0.23–0.80)	0.008	0.037	0.34 (0.13–0.94)	0.037	0.042	0.23 (0.06–0.89)	0.033	0.161	0.90 (0.35–2.30)	0.828	0.638	0.42 (0.13–1.38)	0.153	0.461
Lp(a) < 300 mg/L	0.92 (0.66–1.28)	0.629		0.95 (0.66–1.36)	0.771		1.08 (0.64–1.83)	0.779		1.04 (0.48–2.25)	0.920		0.92 (0.53–1.58)	0.752		0.88 (0.46–1.68)	0.693	
PCSK9																		
PCSK9 ≥ 47.4 ng/mL	0.60 (0.41–0.87)	0.007	0.036	0.61 (0.41–0.92)	0.019	0.104	0.66 (0.36–1.20)	0.171	0.261	0.54 (0.23–1.30)	0.170	0.782	0.75 (0.41–1.38)	0.354	0.403	0.65 (0.32–1.33)	0.236	0.387
PCSK9 < 47.4 ng/mL	0.99 (0.65–1.50)	0.955		0.85 (0.54–1.35)	0.491		1.17 (0.58–2.37)	0.663		0.77 (0.28–2.14)	0.623		0.96 (0.48–1.94)	0.915		0.78 (0.34–1.78)	0.562	
LDL-C																		
LDL-C ≥ 2.6 mmol/L	0.78 (0.51–1.19)	0.246	0.819	0.74 (0.46–1.19)	0.215	0.555	0.98 (0.45–2.13)	0.955	0.454	0.59 (0.17–2.01)	0.402	0.781	0.99 (0.47–2.05)	0.970	0.511	0.63 (0.28–1.40)	0.259	0.968
LDL-C < 2.6 mmol/L	0.71 (0.49–1.02)	0.066		0.70 (0.47–1.04)	0.075		0.76 (0.44–1.32)	0.332		0.63 (0.30–1.31)	0.216		0.89 (0.49–1.64)	0.712		0.47 (0.22–1.03)	0.058	
HDL-C																		
HDL-C ≥ 1mmol/L	0.85 (0.60–1.21)	0.372	0.373	0.91 (0.62–1.33)	0.626	0.109	0.68 (0.38–1.20)	0.179	0.611	0.62 (0.28–1.40)	0.251	0.592	1.09 (0.61–1.94)	0.771	0.279	0.82 (0.42–1.59)	0.548	0.195
HDL-C < 1mmol/L	0.68 (0.44–1.07)	0.096		0.60 (0.37–0.99)	0.044		0.91 (0.40–2.05)	0.820		0.46 (0.12–1.76)	0.256		0.62 (0.29–1.30)	0.206		0.45 (0.18–1.11)	0.082	
TG																		
TG ≥ 1.7 mmol/L	0.91 (0.57–1.47)	0.704	0.992	0.97 (0.58–1.62)	0.918	0.520	0.77 (0.29–2.02)	0.594	0.755	1.05 (0.24–4.57)	0.945	0.494	1.12 (0.52–2.45)	0.771	0.582	0.59 (0.24–1.43)	0.240	0.622
TG < 1.7 mmol/L	0.76 (0.53–1.07)	0.117		0.69 (0.47–1.03)	0.069		0.92 (0.55–1.52)	0.740		0.76 (0.37–1.57)	0.460		0.84 (0.46–1.54)	0.583		0.66 (0.33–1.34)	0.249	

CI = confidence interval, HDL-C = high-density lipoprotein cholesterol,HR = hazard ratio, LDL-C = low-density lipoprotein cholesterol, lp(a) = lipoprotein(a), MACE = major adverse cardiovascular events, PCSK9 = proprotein convertase subtilisin/kexin type 9, P int = P for interaction, re-MI = recurrent MI, TG = triglyceride.

Supplementary Table S8. Interactions between LL-37, PCSK9 and lp(a) for the outcome of MACE.

Variable/term	HR (95% CI) *	P-value
LL-37 (high vs low)	1.25 (0.82–1.89)	0.298
lp[a] (≥ 300 vs < 300 mg/L)	1.17 (0.81–1.69)	0.397
LL-37#lp[a]	0.55 (0.31–0.97)	0.040
PCSK9 (≥ 47.4 vs < 47.4 ng/mL)	1.59 (1.14–2.23)	0.007
LL-37#PCSK9	0.58 (0.34–0.96)	0.036

* Adjusted for age, gender, body mass index, hypertension, previous myocardial infarction, heart rate, ejection fraction, leucocytes, hemoglobin, creatinine, hemoglobin A1c, high-sensitivity C-reactive protein, cardiac troponin I, N-terminal prohormone of brain natriuretic peptide, low-density lipoprotein cholesterol, glucose, culprit lesion, multivessel disease, successful revascularization, P2Y12 inhibitor, statin. CI = confidence interval, HR = hazard ratio, lp(a) = lipoprotein(a), MACE = major adverse cardiovascular events, PCSK9 = proprotein convertase subtilisin/kexin type 9.

Supplementary Table S9. Associations between LL-37 and MACE risk stratified by combinations of lp(a) and PCSK9.

Stratifications	HR (95% CI) *	P-value	$P_{interaction}$
lp(a) ↓ /PCSK9 ↓	1.30 (0.81–2.10)	0.283	0.030
lp(a) ↓ /PCSK9 ↑	0.65 (0.41–1.02)	0.062	
lp(a) ↑ /PCSK9 ↓	0.42 (0.16–1.13)	0.085	
lp(a) ↑ /PCSK9 ↑	0.45 (0.21–0.95)	0.036	

* Adjusted for age, gender, body mass index, hypertension, previous myocardial infarction, heart rate, ejection fraction, leucocytes, hemoglobin, creatinine, hemoglobin A1c, high-sensitivity C-reactive protein, cardiac troponin I, N-terminal prohormone of brain natriuretic peptide, low-density lipoprotein cholesterol, glucose, culprit lesion, multivessel disease, successful revascularization, P2Y12 inhibitor, statin. ↓ = low, ↑ = high, CI = confidence interval, HR = hazard ratio, lp(a) = lipoprotein(a), MACE = major adverse cardiovascular events, PCSK9 = proprotein convertase subtilisin/kexin type 9.

Supplementary Figure S1. Clinical outcomes according to levels of lp(a) and LL-37, including (A) MACE, (B) cardiac death, recurrent MI or ischemic stroke, (C) all-cause death, (D) cardiac death, (E) recurrent MI, and (F) ischemic stroke. lp(a) = lipoprotein (a), MACE = major adverse cardiovascular event, MI = myocardial infarction, ↓ low, ↑ = high.

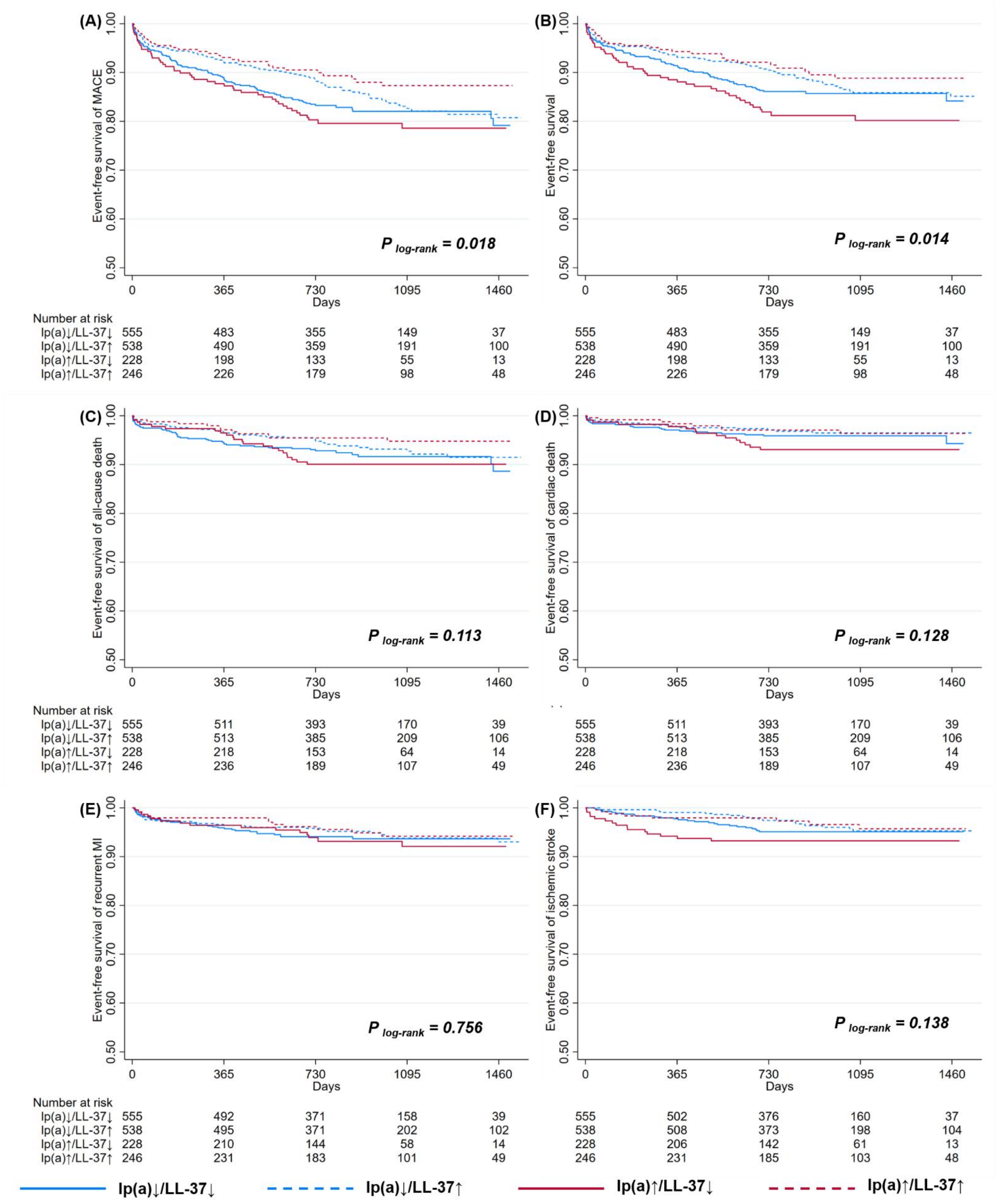

Supplementary Figure S2. Clinical outcomes according to levels of PCSK9 and LL-37, including (A) MACE, (B) cardiac death, recurrent MI or ischemic stroke, (C) all-cause death, (D) cardiac death, (E) recurrent MI, and (F) ischemic stroke. PCSK9 = proprotein convertase subtilisin/kexin type 9, MACE = major adverse cardiovascular event, MI = myocardial infarction, ↓ = low, ↑ = high.

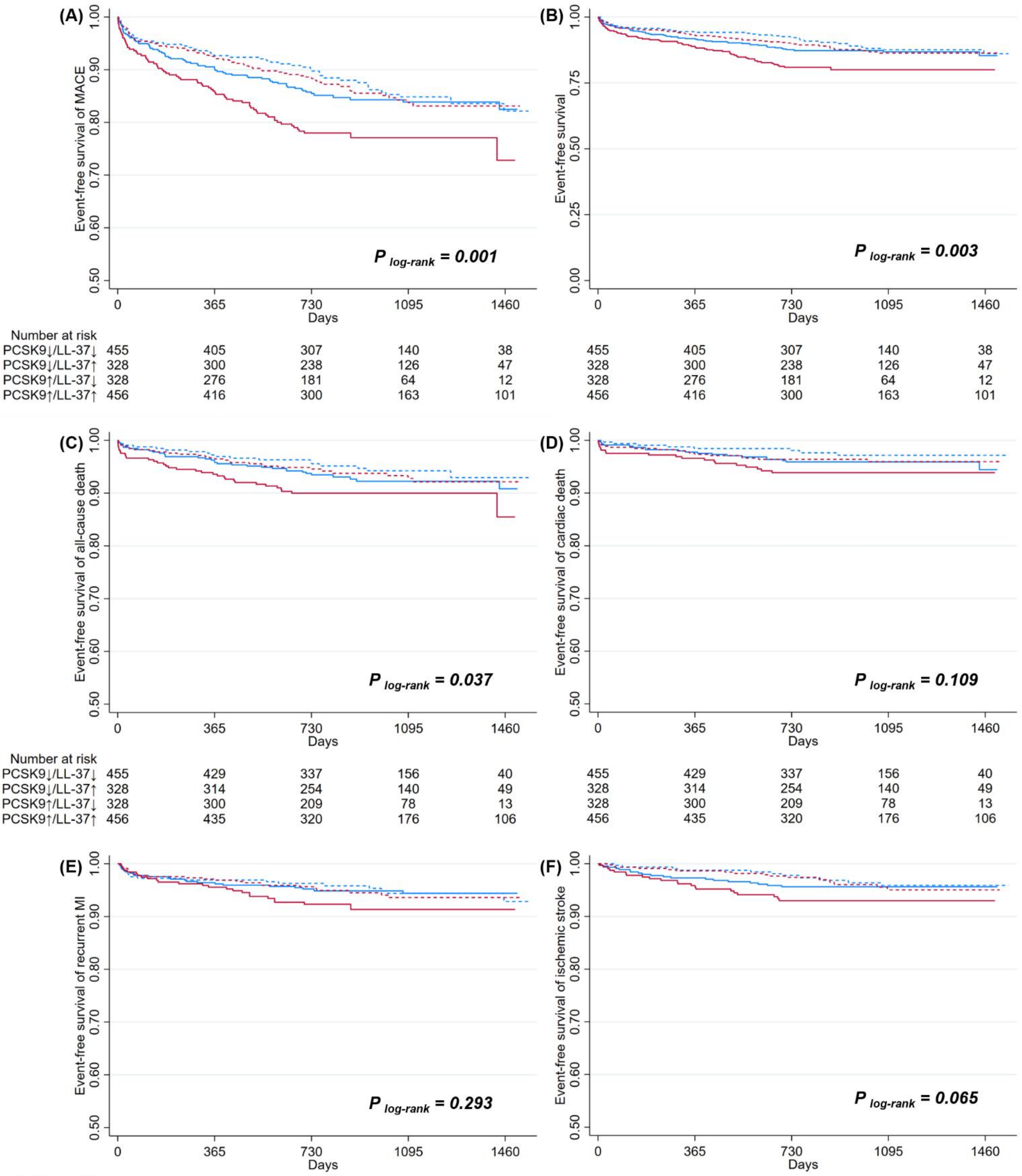

Number at risk (A, B)	0	365	730	1095	1460
PCSK9↓/LL-37↓	455	405	307	140	38
PCSK9↓/LL-37↑	328	300	238	126	47
PCSK9↑/LL-37↓	328	276	181	64	12
PCSK9↑/LL-37↑	456	416	300	163	101

Number at risk (C, D)	0	365	730	1095	1460
PCSK9↓/LL-37↓	455	429	337	156	40
PCSK9↓/LL-37↑	328	314	254	140	49
PCSK9↑/LL-37↓	328	300	209	78	13
PCSK9↑/LL-37↑	456	435	320	176	106

Number at risk (E)	0	365	730	1095	1460
PCSK9↓/LL-37↓	455	415	321	147	40
PCSK9↓/LL-37↑	328	304	245	132	47
PCSK9↑/LL-37↓	328	287	194	69	13
PCSK9↑/LL-37↑	456	422	309	171	104

Number at risk (F)	0	365	730	1095	1460
PCSK9↓/LL-37↓	455	419	322	148	38
PCSK9↓/LL-37↑	328	310	247	134	49
PCSK9↑/LL-37↓	328	289	196	73	12
PCSK9↑/LL-37↑	456	429	311	167	103

PCSK9↓/LL-37↓ PCSK9↓/LL-37↑ PCSK9↑/LL-37↓ PCSK9↑/LL-37↑

Supplementary Figure S3. Clinical outcomes according to levels of LDL-C and LL-37, including (A) MACE, (B) cardiac death, recurrent MI or ischemic stroke, (C) all-cause death, (D) cardiac death, (E) recurrent MI, and (F) ischemic stroke. LDL-C = low-density lipoprotein cholesterol, MACE = major adverse cardiovascular event, MI = myocardial infarction, ↓ = low, ↑ = high.

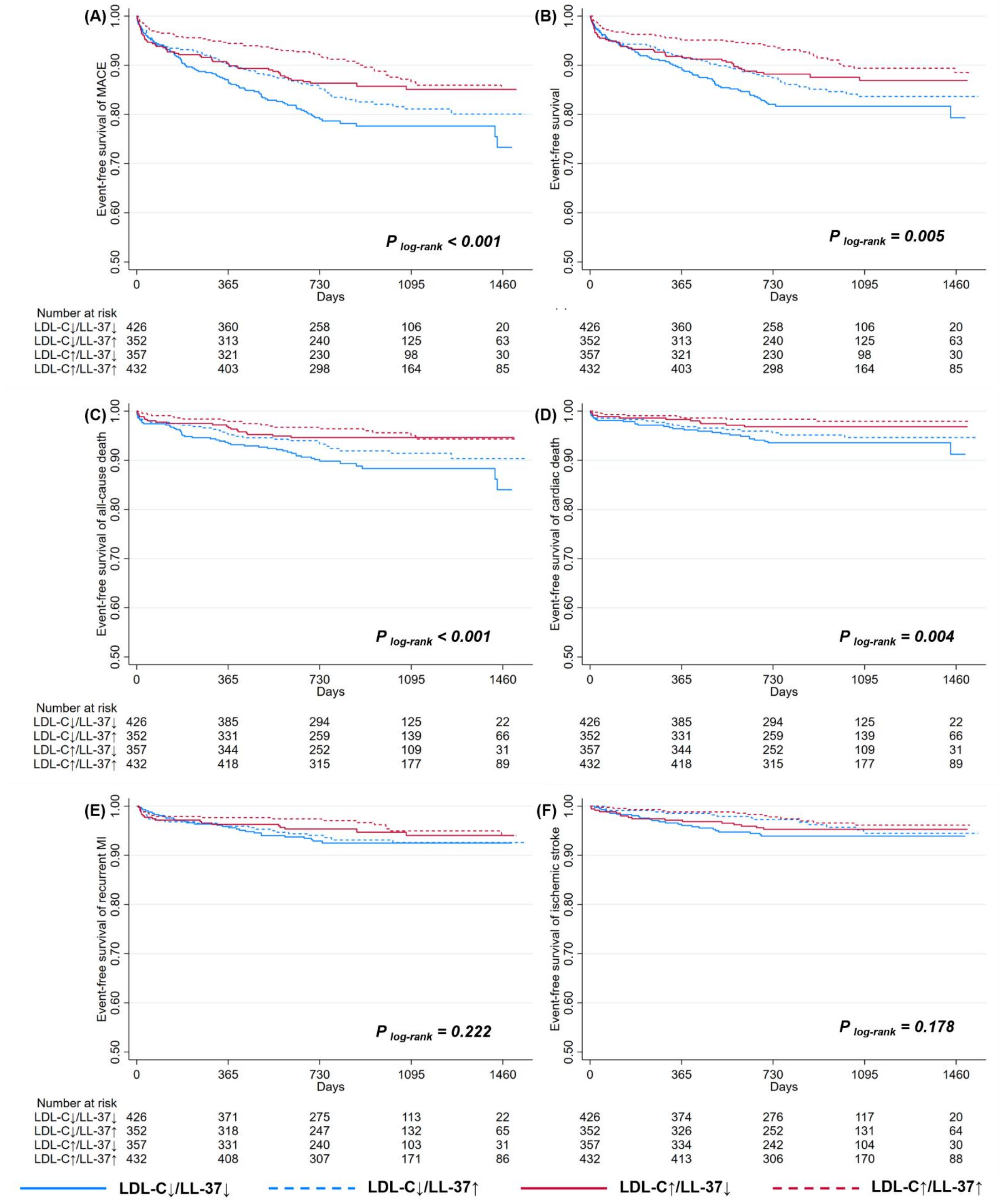

Supplementary Figure S4. Clinical outcomes according to levels of HDL-C and LL-37, including ((A) MACE, (B) cardiac death, recurrent MI or ischemic stroke, (C) all-cause death, (D) cardiac death, (E) recurrent MI, and (F) ischemic stroke. HDL-C = high-density lipoprotein cholesterol, MACE = major adverse cardiovascular event, MI = myocardial infarction, ↓ = low, ↑ = high.

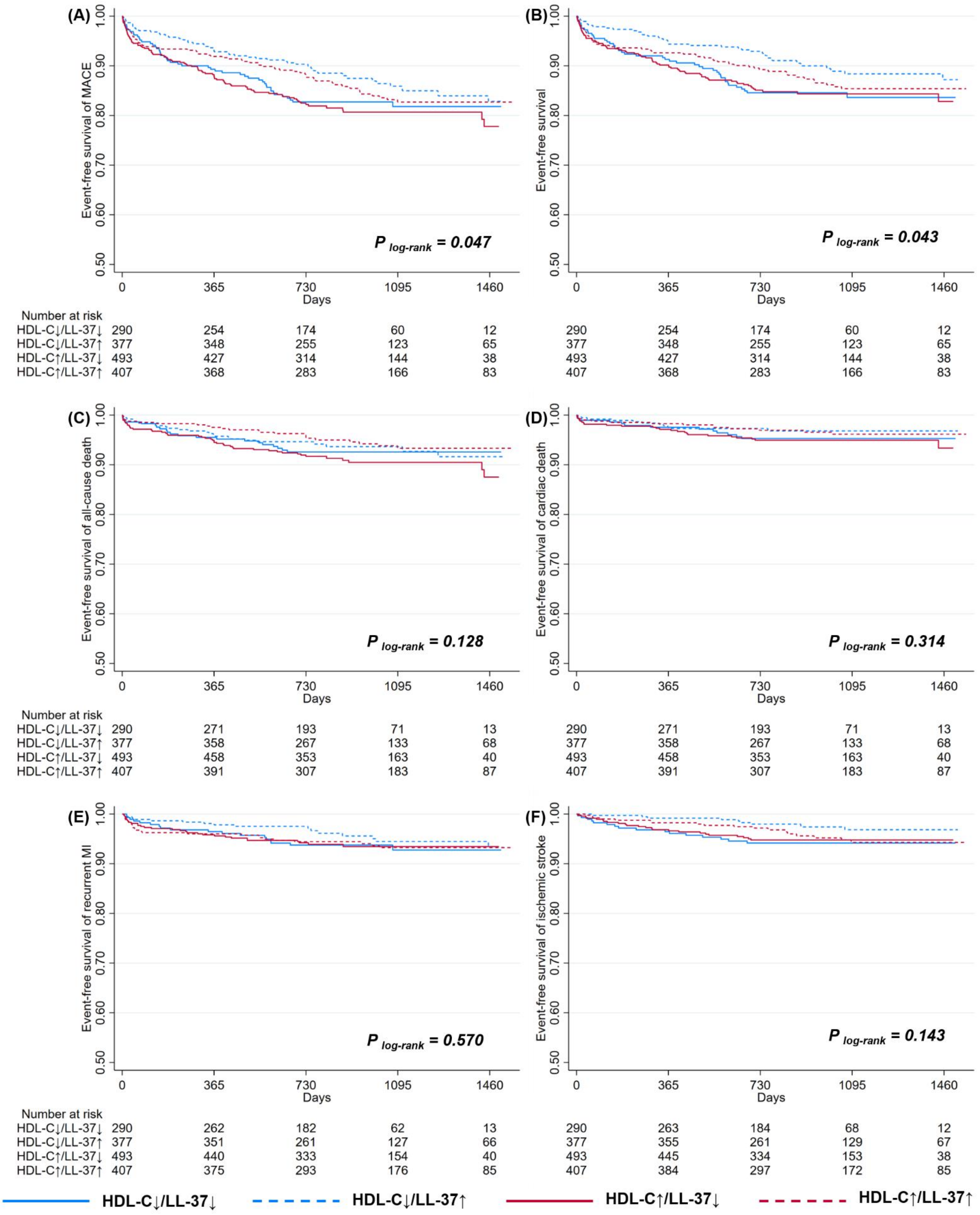

Supplementary Figure S5. Clinical outcomes according to levels of TG and LL-37, including (A) MACE, (B) cardiac death, recurrent MI or ischemic stroke, (C) all-cause death, (D) cardiac death, (E) recurrent MI, and (F) ischemic stroke. TG = triglyceride, MACE = major adverse cardiovascular event, MI = myocardial infarction, ↓ = low, ↑ = high.

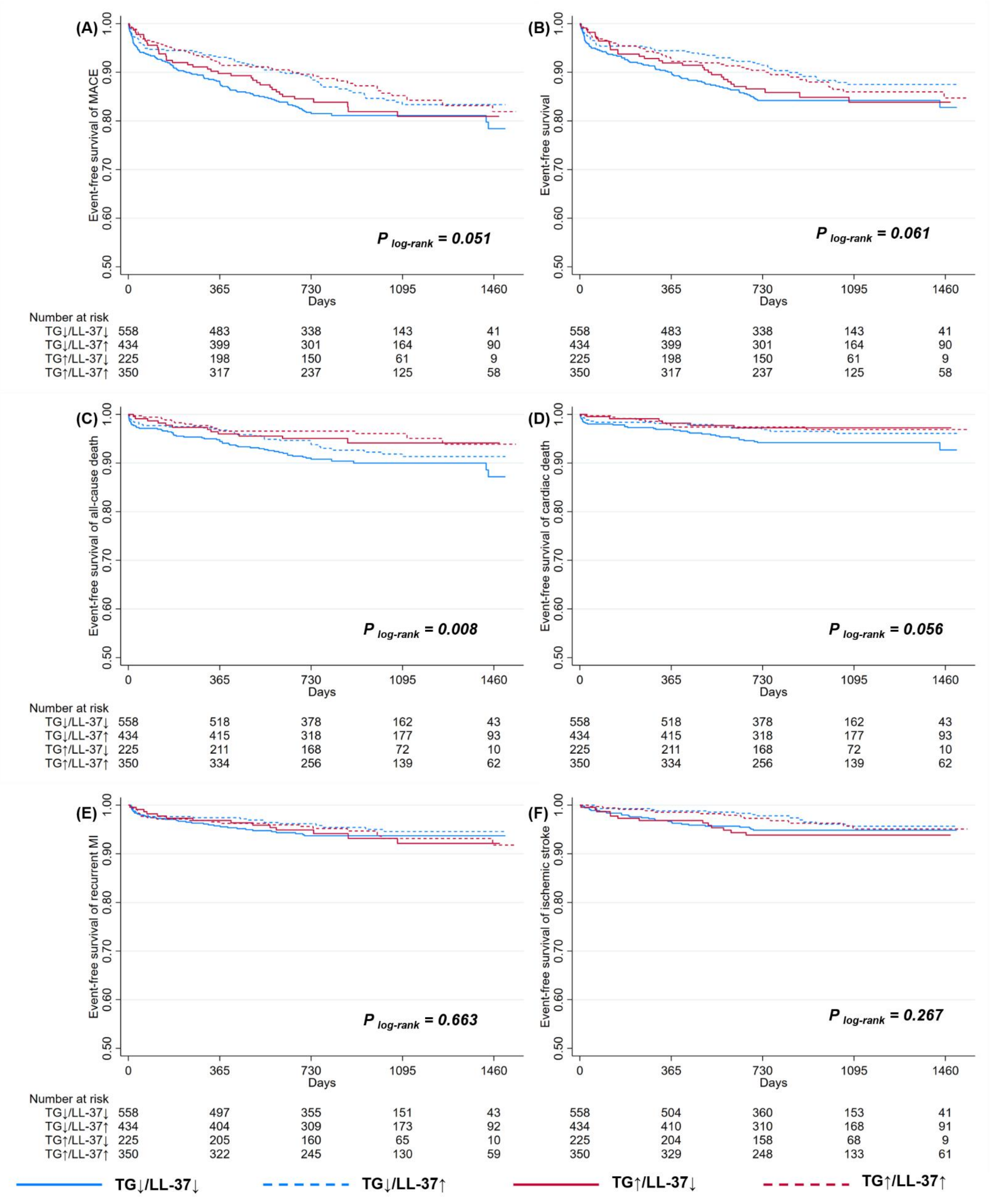

Supplementary Figure S6. Survival curve analysis adjusted for age, gender and body mass index according to levels of lp(a) and LL-37, including (A) MACE, (B) cardiac death, recurrent MI or ischemic stroke, (C) all-cause death, (D) cardiac death, (E) recurrent MI, and (F) ischemic stroke. lp(a) = lipoprotein(a), MACE = major adverse cardiovascular event, MI = myocardial infarction, ↓ low, ↑ = high.

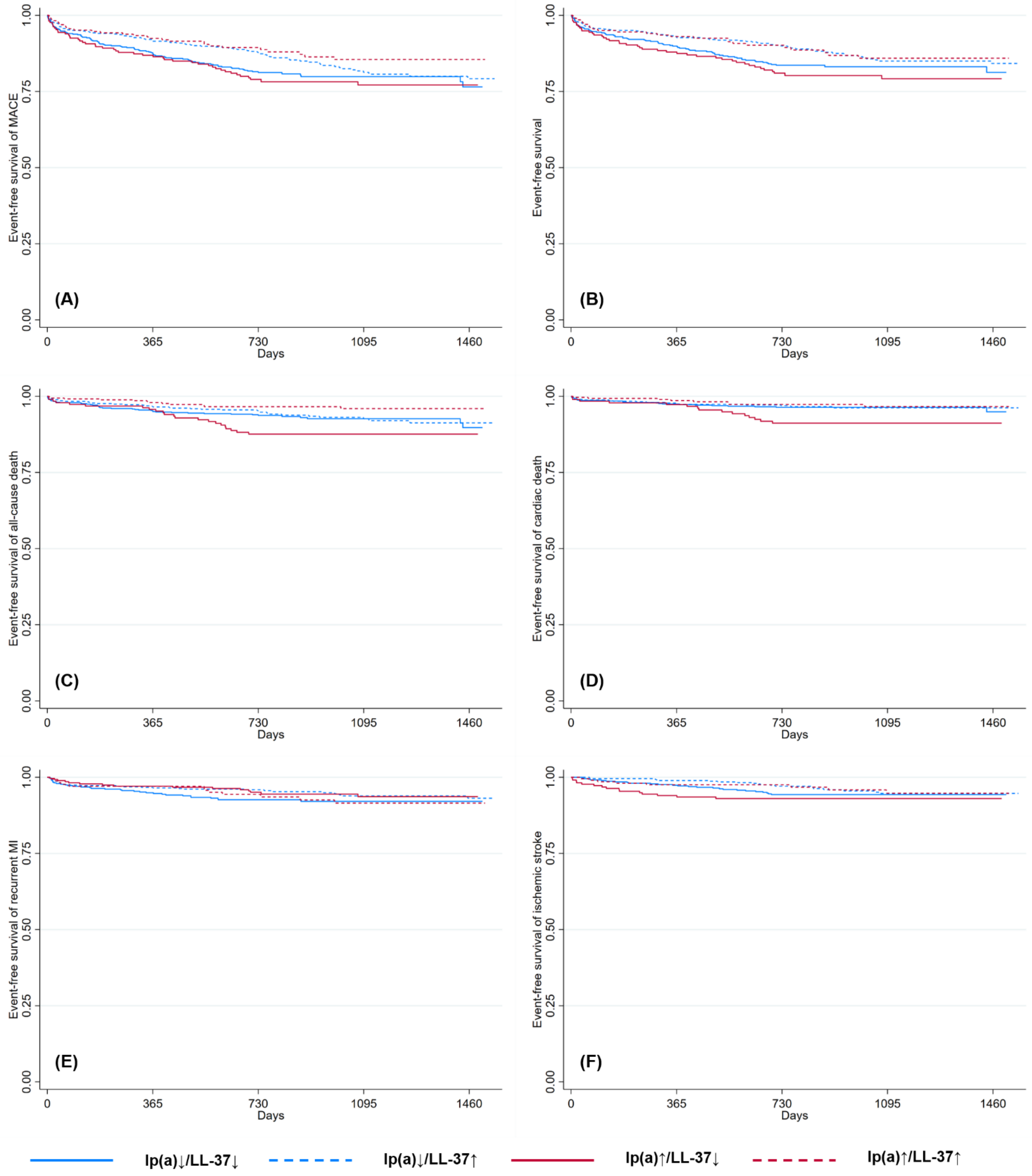

Supplementary Figure S7. Survival curve analysis adjusted for age, gender and body mass index according to levels of PCSK9 and LL-37, including (A) MACE, (B) cardiac death, recurrent MI or ischemic stroke, (C) all-cause death, (D) cardiac death, (E) recurrent MI, and (F) ischemic stroke. PCSK9 = proprotein convertase subtilisin/kexin type 9, MACE = major adverse cardiovascular event, MI = myocardial infarction, ↓ = low, ↑ = high.

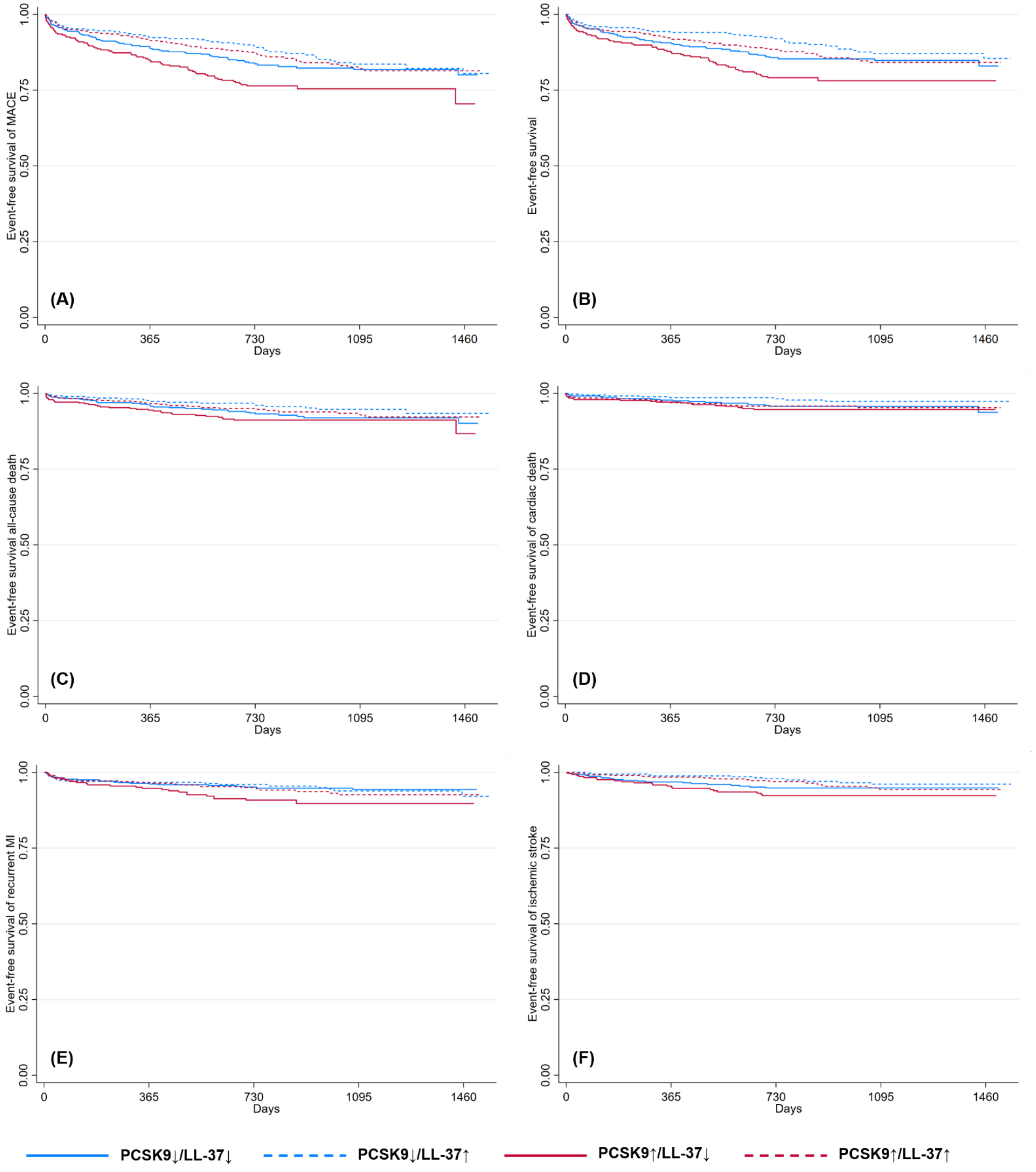

Supplementary Figure S8. Survival curve analysis adjusted for age, gender and body mass index according to levels of LDL-C and LL-37, including (A) MACE, (B) cardiac death, recurrent MI or ischemic stroke, (C) all-cause death, (D) cardiac death, (E) recurrent MI, and (F) ischemic stroke. LDL-C = low-density lipoprotein cholesterol, MACE = major adverse cardiovascular event, MI = myocardial infarction, ↓ = low, ↑ = high.

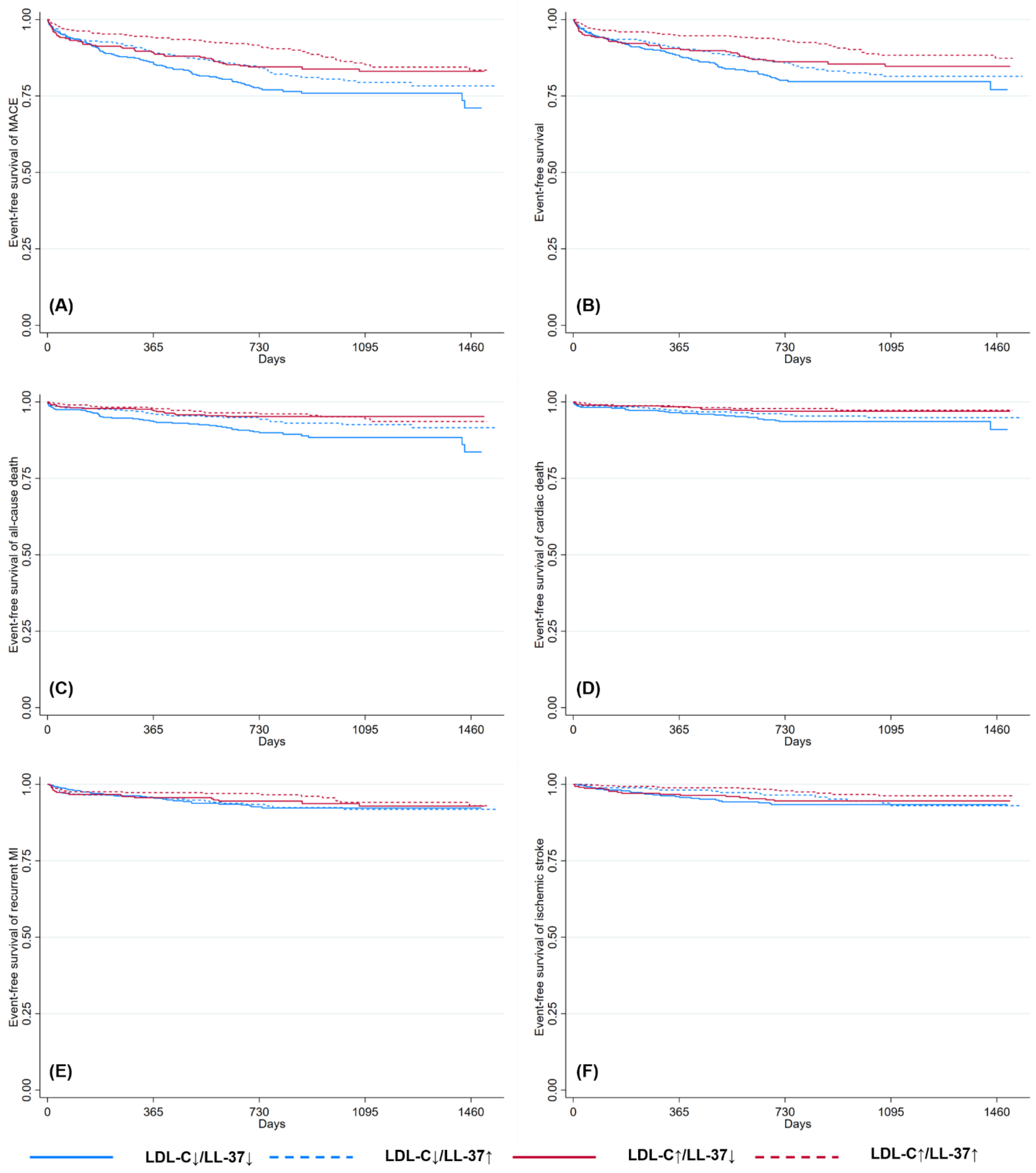

Supplementary Figure S9. Survival curve analysis adjusted for age, gender and body mass index according to levels of HDL-C and LL-37, including (A) MACE, (B) cardiac death, recurrent MI or ischemic stroke, (C) all-cause death, (D) cardiac death, (E) recurrent MI, and (F) ischemic stroke. HDL-C = high-density lipoprotein cholesterol, MACE = major adverse cardiovascular event, MI = myocardial infarction, ↓ = low, ↑ = high.

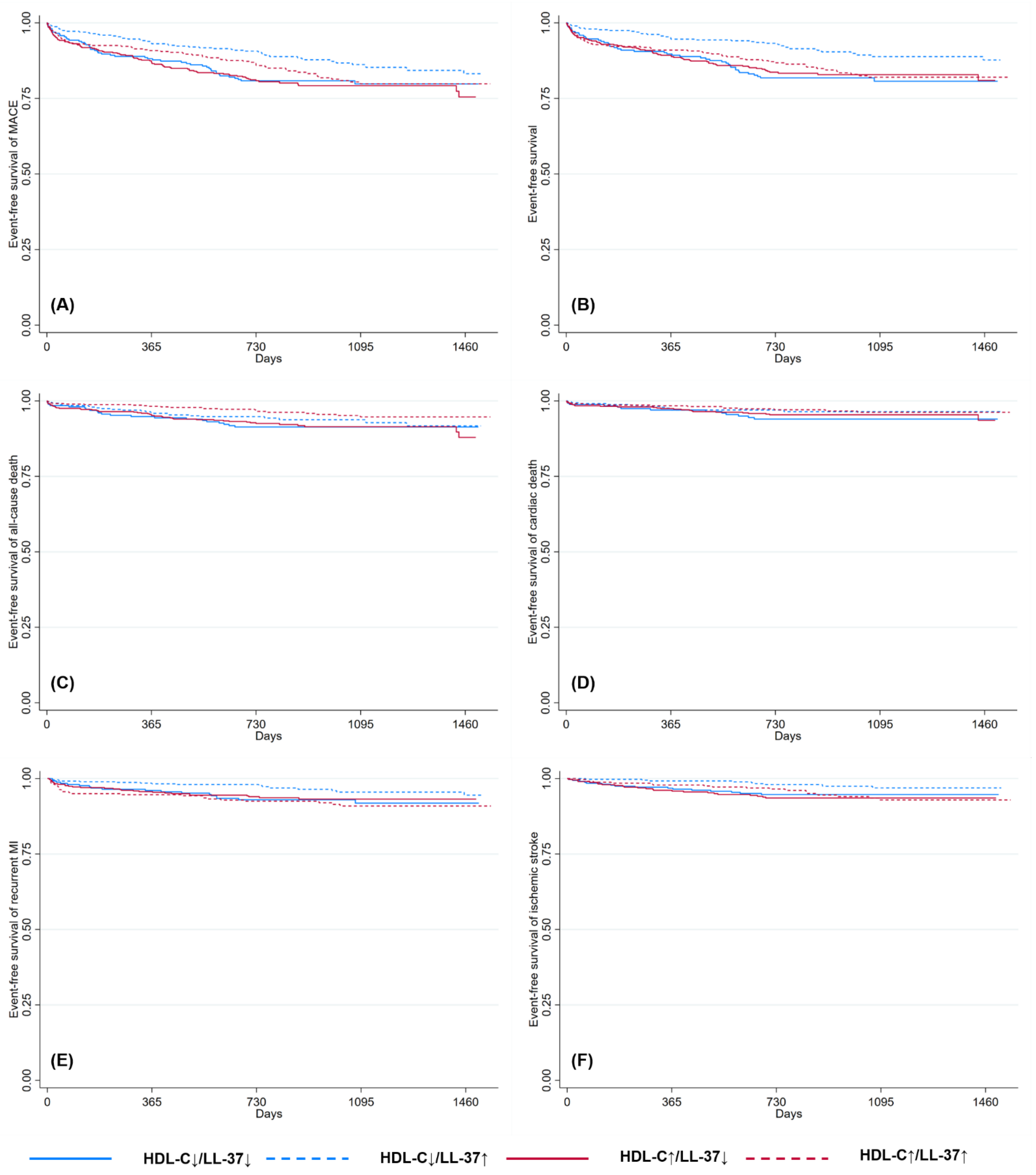

Supplementary Figure S10. Survival curve analysis adjusted for age, gender and body mass index according to levels of TG and LL-37, including (A) MACE, (B) cardiac death, recurrent MI or ischemic stroke, (C) all-cause death, (D) cardiac death, (E) recurrent MI, and (F) ischemic stroke. TG = triglyceride, MACE = major adverse cardiovascular event, MI = myocardial infarction, ↓ = low, ↑ = high.

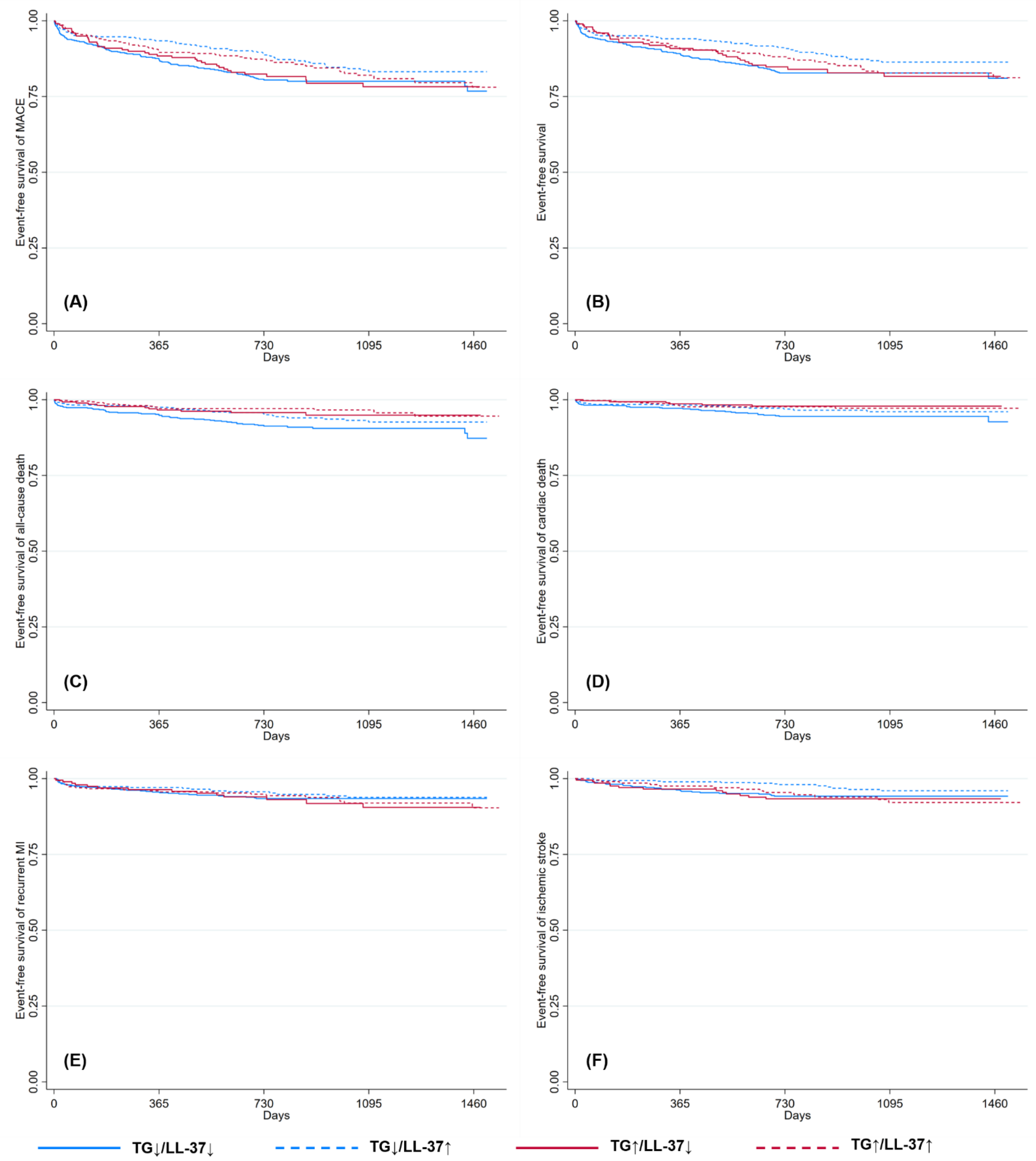

Supplement: Supplementary file 1 [file biomolecules-12-01482-s001.zip › biomolecules-1909800-supplementary.pdf]
